# Pulmonary artery pseudoaneurysm after a left upper sleeve lobectomy

**DOI:** 10.1186/1477-7819-11-272

**Published:** 2013-10-13

**Authors:** Minwei Bao, Yiming Zhou, Gening Jiang, Chang Chen

**Affiliations:** 1 Department of General Thoracic Surgery, Shanghai Pulmonary Hospital, Tongji University School of Medicine, Shanghai, 200433, China

**Keywords:** Aneurysm, Lobectomy, Reoperation, Trauma

## Abstract

A 55-year-old man was re-admitted for persistent hemoptysis and high fever three weeks after an initial left upper sleeve lobectomy for a central squamous lung cancer tumor. Pulmonary artery pseudoaneurysm and pulmonary infection were confirmed by multidetector computed tomography angiography and subsequent emergency completion pneumonectomy. The development of pulmonary artery pseudoaneurysm, secondary to post-operative pulmonary infection and pulmonary vascular manipulation, is rare and prompt surgical manipulation is mandatory.

## Background

Pulmonary artery pseudoaneurysm (PAP) is a rare but potentially lethal postoperative complication. In total, there have been no more than 50 such cases documented on PubMed, among which, only two cases have evolved after right lung lobectomy [1,2]. We herein report a case of a PAP following a left upper sleeve lobectomy for a central squamous lung cancer tumor.

## Case presentation

A 55-year-old man initially presented to our department with a 6-month refractory dry cough and bloody sputum. Physical examination and past medical history were unremarkable. Chest computed tomography (CT) showed a lesion in the left upper lobe with prominent obstructive lobar pneumonitis. Bronchoscopy revealed a neoplasm on the left superior lobar bronchus and mucosal swelling that extended to the distal end of the left main bronchus. Tissue biopsy confirmed a squamous cell carcinoma and sleeve lobectomy was scheduled after excluding remote metastasis.

A routine sleeve lobectomy was performed via open thoracotomy. Three rings of the distal left lower main bronchus were removed and end-to-end bronchial reconstruction was performed after confirming negative margins by frozen section. The bronchial anastomosis was enveloped tightly with a pedicled parietal pleura. Mediastinal nodal dissection was also completed and included stations 4–9. However, when the anesthesiologist inflated the remnant lung, we noticed an obviously tortuous pulmonary artery due to a lengthy resection of the main bronchus. Therefore, we decided to shorten the artery correspondingly. Considering that a sleeve resection of the pulmonary artery was time-consuming, a latitude-direction enfolding was performed with a continuous running suture with a 5–0 Prolene. Meticulous inspection of the pulmonary artery found neither sub-mucous hematoma nor occlusion or narrowing of the artery. The patient recovered well and was discharged on the ninth post-operative day. The final pathology confirmed squamous cell carcinoma of the left upper lung at stage p-T2N0M0 IB.

Unfortunately, the patient experienced a persistently high fever over 39°C with dark-yellow sputum five days after discharge and mild hemoptysis another two days later. A chest CT examination at a local hospital revealed obvious left pneumonia with moderate pleural effusion. No artery abnormality was reported. Following, imipenem was administered and symptoms of infection resolved one week later; however, the symptom of hemoptysis prolonged and the patient was re-admitted to our hospital.

**Figure 1:**
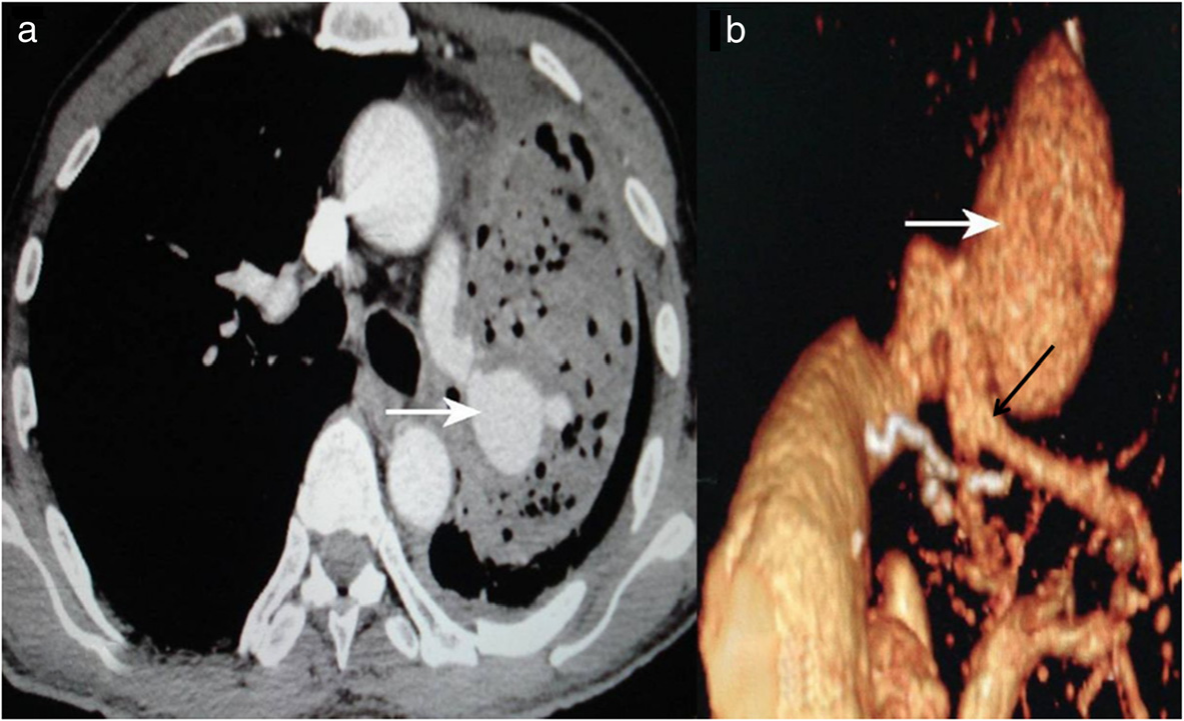
**Three-dimensional (3-D) radiography confirmed a pseudoaneurysm feeding from the dorsal segment branch of the left lower lobe with apparent proximal arterial enlargement.** The pseudoaneurysm (white arrow) was 3.2 × 2.8 cm in size and surrounded by infected thrombus, pleural effusion, and lung tissue **(a)**. The pseudoaneurysm was also apparently huge compared to the feeding pulmonary artery under 3-D imaging **(b**, white arrow**)**. The angioplasty lesion was indicated by a black arrow **(b)**.

Physical examination upon admission revealed rales at the base of the left lung; no murmur was heard. A multidetector computed tomography angiography (MDCTA) scan showed a left pulmonary pseudoaneurysm approximately 3.2 × 2.8 cm in size, which was embedded within circumferential pleural effusion and adjacent infected pulmonary tissue. Three-dimensional (3-D) radiography confirmed a pseudoaneurysm feeding from the dorsal segment branch of the left lower lobe with apparent proximal arterial enlargement (Figure 1). A bronchoscopic examination indicated good bronchial anastomosis and, therefore, excluded the suspicion of bronchopleural fistula. A constant bloody purulent effusion was prominently visible from the dorsal segment bronchus of the left lower lobe. Considering the lethal risk of pseudoaneurysm rupture, we performed an emergency completion pneumonectomy.

There was intense adhesion all over the pleural space. Most of the remnant left lower lung was densely consolidated. Dissection of the pulmonary artery trunk was extremely difficult because of the intense adhesion around the aortic window. The pericardium was then opened to free the pulmonary artery trunk; however, the aneurysm ruptured under compression. The left artery trunk had to be closed proximal to the ligamenta arteriosum pulmonalis.

**Figure 2:**
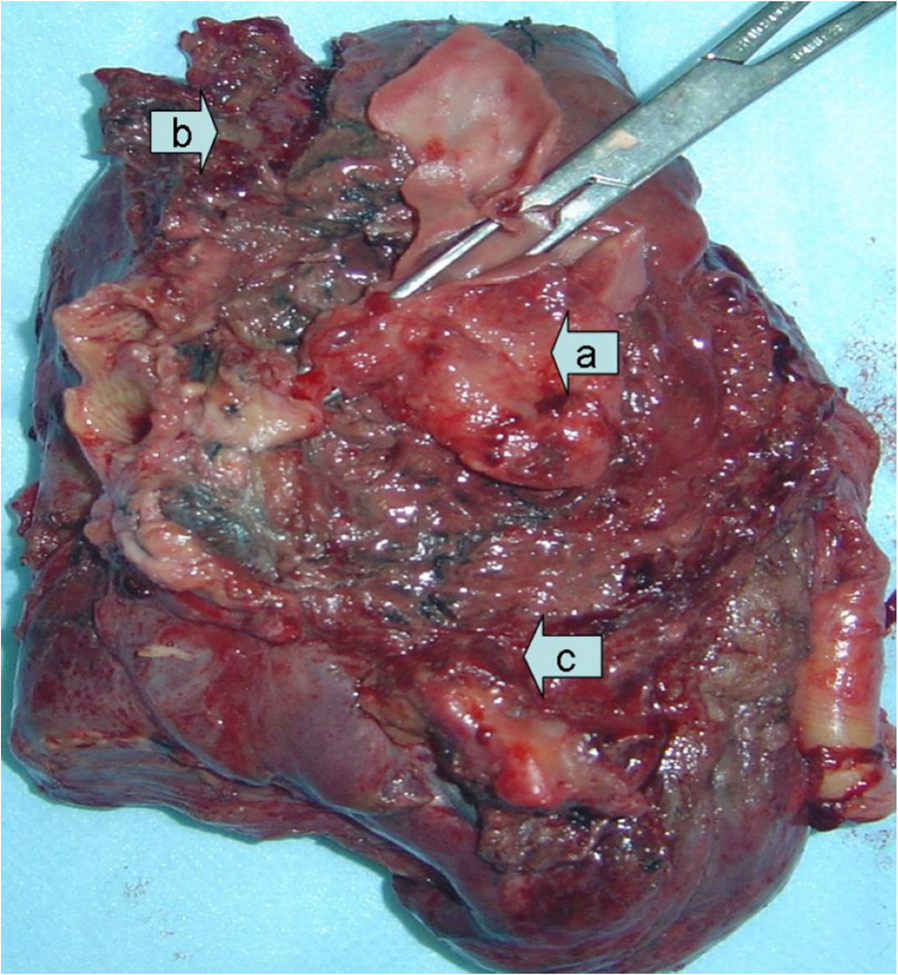
Macroscopically, the pseudoaneurysm (a) was surrounded by a massive infected hematoma (b) and largely destroyed remnant left lung tissue (c).

Macroscopic inspection of the specimen revealed a ruptured pseudoaneurysm abutting into the infected hematoma as well as consolidated and largely destroyed remnant left lung tissue (Figure 2). The effusion from the infected tissue released an odor. Pathology demonstrated an infected left lower lobe and pulmonary pseudoaneurysm. A culture of the infected pulmonary tissue showed growth of *Leuconostoc mesenteroides* subsp. and *Streptococcus mitis*, which were both multi-resistant to ceftriaxone sodium and levofloxacin.

The patient developed postoperative empyema. Pleural lavage with iodine (1:10 diluted, 1,000 mL, twice daily) was administered for 26 continuous days; however, this treatment failed as evidenced by repeated bacteria retention in the pleural effusion. Of note, the empyema eventually healed after 46 days with chest tube water-sealed drainage. No further chemotherapies were administered. Follow-up revealed a normal quality of life and a cancer-free status one year after the first operation for lung cancer.

## Conclusions

PAP is an uncommon but life-threatening condition; a variety of causes have been proposed concerning its etiology and most suggest pulmonary pseudoaneurysm is acquired [3]. PAP often occurs after chest trauma, from pulmonary infection, or because of iatrogenic reasons (e.g., interventional treatment) [3]. McQueen et al. proposed that iatrogenic trauma is the most common reason [4]. In the present case, both intrathoracic infection and iatrogenic trauma to the artery were equally contributive.

All cases face life-threatening risks of hemoptysis and suffocation, which makes prompt diagnosis and intervention mandatory to reduce the life threatening risk and save a patient’s life [3-5]. The gold standard for diagnosis of PAP is pulmonary angiography, but this is now largely superseded by non-invasive CT angiography and 3-D reconstruction. In this study, we used MDCTA and 3-D radiography to diagnose the PAP. For early detection of the PAP, we suggest to perform contrast-enhanced CT or pulmonary angiography after sleeve lobectomy.

Hemoptysis is the most frequent and life threatening symptom after pulmonary artery rupture [6,7]. In the present case, hemoptysis occurred one week after discharge. Fortunately, the intense and extensive pleural adhesions largely limited the expansion and explosion of the pseudoaneurysm into the chest cavity, which would have been otherwise fatal in the early stage of disease development. Treatment of PAP can be surgical through aneurysmectomy and/or lobectomy, or radiological through steel or tungsten coil embolization [1]. In our case, emergency completion pneumonectomy over transcatheter coil embolization management [2] was selected because of the extensive pulmonary and pleural infection and the grossly consolidated lung. Coil placement may result in luminal obstruction of the left pulmonary artery, and subsequently, lead to lung infarction.

## Consent

Written informed consent was obtained from the patient for publication of this Case Report and any accompanying images. A copy of the written consent is available for review by the Editor-in-Chief of this journal.

## Abbreviations

MDCTA: Multidetector computed tomography angiography; PAP: Pulmonary artery pseudoaneurysm.

## Competing interests

The authors declare that they have no competing interests.

## Authors’ contributions

MWB designed research; YMZ performed research; GNT analyzed data; CC wrote the paper. All authors read and approved the final manuscript.

## References

[B1] Shaaban H Sharma H Rao J Clark S A pulmonary artery false aneurysm after right middle lobectomy: a case report J Med Case Rep 2007 11 70 10.1186/1752-1947-1-70 17718925 PMC2014767

[B2] Matsumura Y Shiono S Saito K Sato T Pulmonary artery pseudoaneurysm after lung resection successfully treated by coil embolization Interact Cardiovasc Thorac Surg 2010 11 364 365 10.1510/icvts.2010.236661 20515920

[B3] Lafita V Borge MA Demos TC Pulmonary artery pseudoaneurysm: etiology, presentation, diagnosis, and treatment Seminars in Interventional Radiology 2007 Stuttgart: Thieme Medical Publishers 119 10.1055/s-2007-971202 PMC3036336 21326750

[B4] McQueen A Mitchell L Muller M MacGowan G Corris P Iatrogenic pulmonary artery pseudoaneurysm: images from different modalities Thorax 2008 11 938 938 10.1136/thx.2007.085324 18820125

[B5] Nguyen ET Silva CIS Seely JM Chong S Lee KS Müller NL Pulmonary artery aneurysms and pseudoaneurysms in adults: findings at CT and radiography Am J Roentgenol 2007 11 W126 W134 10.2214/AJR.05.1652 17242217

[B6] Sbano H Mitchell AW Ind PW Jackson JE Peripheral pulmonary artery pseudoaneurysms and massive hemoptysis AJR Am J Roentgenol 2005 11 1253 1259 10.2214/ajr.184.4.01841253 15788606

[B7] Bussieres JS Iatrogenic pulmonary artery rupture Curr Opin Anaesthesiol 2007 11 48 52 10.1097/ACO.0b013e32801158a9 17211167

